# Reduced Glutamine Synthetase Activity Alters the Fecundity of Female *Bactrocera dorsalis* (Hendel)

**DOI:** 10.3390/insects10070186

**Published:** 2019-06-27

**Authors:** Dong Wei, Meng-Yi Zhang, Ying-Xin Zhang, Su-Yun Zhang, Guy Smagghe, Jin-Jun Wang

**Affiliations:** 1 Chongqing Key Laboratory of Entomology and Pest Control Engineering, College of Plant Protection, Southwest University, Chongqing 400716, China; 2 International Joint Laboratory of China-Belgium on Sustainable Crop Pest Control, State Cultivation Base of Crop Stress Biology for Southern Mountainous Land, Academy of Agricultural Sciences, Southwest University, Chongqing 400716, China; 3 Department of Plants and Crops, Faculty of Bioscience Engineering, Ghent University, Ghent 9000, Belgium

**Keywords:** oriental fruit fly, glutamine synthetase, fecundity, L-methionine S-sulfoximine, vitellogenin

## Abstract

Glutamine synthetase (GS) is a key enzyme in glutamine synthesis and is associated with multiple physiological processes in insects, such as embryonic development, heat shock response, and fecundity regulation. However, little is known about the influence of GS on female fecundity in the oriental fruit fly, *Bactrocera dorsalis*. Based on the cloning of *BdGSs*, mitochondrial *BdGSm* and cytoplasmic *BdGSc*, we determined their expressions in the tissues of adult *B. dorsalis*. *BdGSm* was highly expressed in the fat body, while *BdGSc* was highly expressed in the head and midgut. Gene silencing by RNA interference against two *BdGSs* isoforms suppressed target gene expression at the transcriptional level, leading to a reduced ovarian size and lower egg production. The specific inhibitor L-methionine S-sulfoximine suppressed enzyme activity, but only the gene expression of *BdGSm* was suppressed. A similar phenotype of delayed ovarian development occurred in the inhibitor bioassay. Significantly lower expression of *vitellogenin* and *vitellogenin receptor* was observed when GS enzyme activity was suppressed. These data illustrate the effects of two GS genes on adult fecundity by regulating vitellogenin synthesis in different ways.

## 1. Introduction

Glutamine synthetase (GS, EC 6.3.1.2, L-glutamate: ammonia ligase) is widely distributed in microorganisms, animals, and higher plants, and is involved in many biological processes. It is required to modulate the level of the neurotransmitter glutamate and the level of glutamine biosynthesis. GS is also an essential detoxification enzyme in stress and immune responses [1,2]. Recently, a new role for GS in endothelial cell migration during pathological angiogenesis has been reported in mammals, beyond that of glutamine synthesis [3]. GS is classified into three distinct form: GSI, which is present only in prokaryotes; GSII, found in eukaryotes and some prokaryotes; and GSIII, which is present in anaerobes [4]. In insects, the two types of GS isoforms are mitochondrial GS and cytoplasmic GS, both belonging to the GSII form [5].

The physiological function of both GS isoforms has seldom been reported in insects. In *Drosophila*, mitochondrial GS is necessary in the early stages of embryonic development, and interruption of GS expression causes female sterility [6,7]. In *Aedes aegypti*, *GS* is continuously expressed at all developmental stages [8], and provides the glutamine needed for the initial steps of chitin biosynthesis in the female peritrophic matrix [9]. In *Apis cerana cerana*, *GS* is involved in response to environmental stress, e.g., thermal stress, oxidative stress, and pesticides [2]. In *Acyrthosiphon pisum*, GS works with glutamate synthase (Glts) to incorporate ammonium nitrogen into glutamate. This is a key source of nitrogen, fueling integrated amino acid metabolism in the aphid–*Buchnera* endosymbiont partnership [10]. GS is also an important regulator involved in female *Nilaparvata lugens* fecundity by activating the target of the rapamycin signal pathway [11,12]. The microRNA miR-4868b also plays a crucial role in targeting the *GS* gene in *N. lugens* [11].

The oriental fruit fly, *Bactrocera dorsalis* (Hendel), is one of the most economically important fruit flies. Its high reproductive rate can lead to significant crop losses [13,14]. Two type II *BdGSs* genes, mitochondrial *BdGSm* and cytoplasmic *BdGSc*, have been cloned from *B. dorsalis*, and the function of *BdGSc* in larval development has been studied [15]. Their high expression in adults indicates an important role in the adult stage. In this study, we provide experimental evidences demonstrating that *BdGSm* and *BdGSc* are functionally involved in *B. dorsalis* fecundity.

## 2. Materials and Methods

### 2.1. Insects

A stock colony of *B. dorsalis* was collected as pupae from Haikou, Hainan Province of China, in 2008, and then continuously maintained in our laboratory. The larvae and adults were cultured on an artificial diet at 27.5 ± 0.5 °C, relative humidity of 75 ± 5%, and 14 h light: 10 h dark photoperiod [16]. All insects used for experiments were of the same age.

### 2.2. Gene Expression in Adults

Both *BdGSs* genes, *BdGSm* and *BdGSc*, are expressed at high levels in adult *B. dorsalis* [15]. In this study, we detected their expressions during maturation of the adult stage. Newly emerged adults were caged, and female and male adults were maintained separately. Virgin female and male adults at 0 to 10 d were collected for total RNA isolation using TRIzol reagent (Invitrogen, Carlsbad, CA, USA) according to manufacturer’s instructions. The quality was evaluated by a NanoDrop One instrument (Thermo Fisher Scientific, Madison, WI, USA). After genomic DNA digestion with RQ1 RNase-free DNase (Promega, Madison, WI, USA), the RNA samples were used for first-strand complementary DNA synthesis using a PrimeScript kit (Takara, Dalian, China). The relative expression of both *BdGSs* genes was calculated by a quantitative real-time polymerase chain reaction (qRT-PCR) protocol as used in previous studies [17,18], with a CFX384 Optics Module (Bio-Rad, Singapore). The qRT-PCR reaction was carried out in a 10 μL reaction volume including 5 μL of Novostar-SYBR Supermix (Novoprotein, Shanghai, China), 3.5 μL of nuclease-free water, 0.5 μL of cDNA (400–500 ng/µL), and 0.5 μL each of forward and reverse primers (10 μM). A melting curve analysis from 60 to 95 °C was conducted at the end to ensure the specificity and consistency of all generated products. The fragments of 185 and 150 bp from *BdGSm* and *BdGSc* were selected for qRT-PCR [15]. *Alpha-tubulin* was used as an internal reference gene [19]. Thereafter, the head, thorax, midgut, fat body, Malpighian tubules, and ovary tissues from 9-day-old virgin female adults were dissected in phosphate-buffered solution (PBS, pH = 7.2) for total RNA isolation as above. The relative expressions of both *BdGSs* genes among the adult tissues were also analyzed by qRT-PCR. The gene expression in adults post-eclosion and gene expression among tissues were conducted with three biological replicates, and analyzed by qBase Plus software (Biogazelle, Ghent, Belgium) [20].

### 2.3. RNAi Bioassay

The specific fragments of 497 and 345 bp from the open reading frame of *BdGSm* and *BdGSc* were selected and amplified, respectively. The primers of *BdGSc* were the same as in a previous study [15], and the primers of *BdGSm* were as follows: forward (5’→3’) taatacgactcactatagggGAACCTTGGTTCGGCATTG, reverse (5’→3’) taatacgactcactatagggCCTACGATCCAAAGGAAGG. These fragments show a short overlap with the region for qRT-PCR. Both *BdGSs* gene-specific double-stranded RNAs were synthesized using the Transcript Aid T7 High Yield Transcription Kit (Thermo Scientific, Vilnius, Lithuania). Three batches of females (30 individuals in each) were injected with gene-specific dsRNA of both *BdGSs* genes at 2-, 4-, and 6-day-old three times with 2 μg dsRNA each. Females injected with the same amount of dsGFP were used as a negative control. One batch of injected females was used for total RNA isolation and cDNA preparation as above. The RNAi efficiencies were determined and calculated by qRT-PCR with three biological replicates. The gene expression between control and treatments were analyzed using the 2^−△△Ct^ method [21]. Twenty of the second batch of injected females were dissected in PBS. The ovaries were observed under a binocular stereoscope (KEYENCE, VHX-S550E, Osaka, Japan), and the sizes of each pair of ovaries were calculated according to the mean diameter of each ovary. Thirty of the third batch of injected females were exposed to males of the same age for mating. Ten mated females were randomly selected and separately reared for egg production. Subsequent egg-laying was recorded using a previously described method [14]. Briefly, the orange juice in pored Eppendorf tube was used as the attractant in the container, and the number of eggs was recorded every day. The difference of the RNAi efficiency and the mean number of eggs laid per female individual was analyzed using a Student’s *t*-test (*P* < 0.05) and SPSS 19.0 (IBM, Chicago, IL, USA).

### 2.4. The MSX Bioassay

The GS-specific inhibitor, L-methionine S-sulfoximine (MSX, Sigma, Shanghai, China), which irreversibly blocks the catalytic activity of GS [12], was used to observe the gene expression and enzymatic activity in *B. dorsalis* adult females. Three groups (30 individuals in each) of newly emerged adult females were reared on an artificial diet for two days and then reared with 10 μL/fly of MSX-added solution at three doses of 0.5, 1.0, and 2.5 μM every day. Flies fed on a diet with only purified water added were used as the control. One group of the test female flies was collected after five days of MSX feeding (8-day-old) for gene expression and enzymatic activity determination. The total RNA isolation and RT-qPCR were conducted and analyzed as above to study the gene expression. Enzymatic activity was determined using the GS enzyme reagent kit (Solarbio, Beijing, China) according to manufacturer’s instructions and the methods as used in a previous study [15]. Thirty of the second group of females were collected for ovary dissection in PBS. Ovary diameter was the parameter used to evaluate female ovarian size as described above. The third group of females was collected and allowed to mate with males of the same age. Mating successes were recorded, and the fecundity of the mated females was calculated based on the number of eggs laid for at least five days. The analysis of the ovarian size was performed in the same manner as in the RNAi bioassay. The expressions of two marker genes, *vitellogenin* (*BdVg1*, AF368053) and *vitellogenin receptor* (*BdVgR*, JX469118) [18,22], were thereafter detected by qRT-PCR. Three biological replicates were performed.

### 2.5. Statistical Analysis

The significant differences of both *BdGSs* genes expressions among tissues were evaluated by one-way analysis of variance (ANOVA) with a Turkey LSD using SPSS 19.0 (IBM, Chicago, IL, USA) (*P* < 0.05). The gene expression and enzymatic activity of females feeding with MSX were also analyzed by one-way ANOVA. The significant differences of the RNAi efficiency, the diameter of the ovaries, the mean number of eggs laid per female individual, and two marker genes’ expressions were analyzed using Student’s *t*-test (*P* < 0.05).

## 3. Results

### 3.1. Gene Expression during Sex Maturation

**Figure 1:**
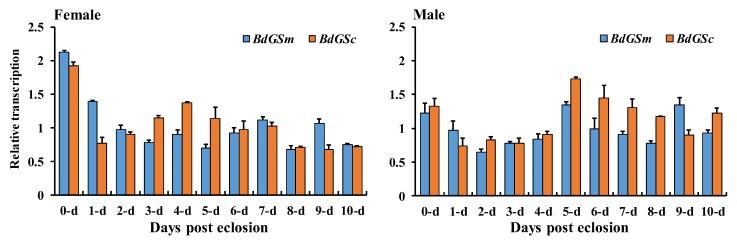
Gene expression of *BdGSm* and *BdGSc* during female and male adult post-eclosion. The gene expression was calculated by qBase Plus software. The bar represents the mean gene expression and the error bar represents the positive standard error of the mean.

**Figure 2:**
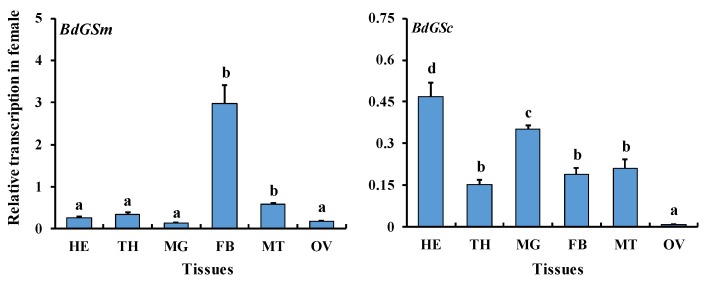
Gene expression of *BdGSm* and *BdGSc* among tissues from 9-day-old female adults. HE, TH, MG, FB, MT, and OV stand for head, thorax, midgut, fat body, Malpighian tubules and ovary, respectively. The gene expression was calculated by qBase Plus software. The bar represents the mean gene expression and the error bar represents the positive standard error of the mean. A different letter on the bar indicates a significant difference analyzed by ANOVA with a Tukey LSD test using SPSS 19.0 (*P* < 0.05).

The gene expression data showed that both *BdGSs* genes were widely expressed in the adult stages (Figure 1). We then determined their expressions in various tissues of 9-day-old female adults. Relative expression profiling showed that *BdGSm* was prominently expressed in the fat body of female adults, while *BdGSc* was highly expressed in most of the tested tissues except for the reproductive tissue (Figure 2). The difference in expression may indicate different roles in *B. dorsalis* female adults.

### 3.2. RNAi Bioassay

**Figure 3:**
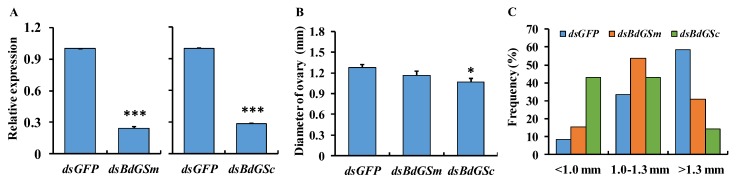
The silencing efficiency for the two *BdGSs* (**A**), ovarian diameter (**B**), and the frequency of the ovarian size (**C**) after three subsequent double-stranded RNA injections. The gene expression in the control group was calculated and normalized to be 1. The bar represents the mean gene expression, the mean ovarian diameter, and the frequency of ovarian size category (*n* = 20). The error bar represents the positive standard error of the mean. The asterisk above the bar indicates the significant difference compared with the control as analyzed by Student’s *t*-test using SPSS 19.0 (* *P* < 0.05; *** *P* < 0.001). The bars in panel C represent the frequency of ovarian size of each category.

We used RNAi to investigate the potential roles of *BdGSm* and *BdGSc* in the female fecundity of *B. dorsalis*. The results indicated that both *BdGSs* genes can be suppressed by gene-specific double-stranded RNA injection. The silencing efficiencies of *BdGSm* and *BdGSc* were 75.4% and 71.8%, respectively, in *B. dorsalis* adults (Figure 3A), after three serial injections with dsRNA at 2-, 4-, and 6-day-old. To investigate their roles in female fecundity, the GS-suppressed females were dissected for ovarian size determination. The average ovarian size of the residual females was smaller in the dsRNA-*BdGSs* group than in the control group (*P* = 0.015, Figure 3B). The frequency of ovarian size differed between categories. In the control, 60% of the ovaries were >1.3 mm, while 30% were >1.3 mm in the *BdGSm*-silenced group, and only 15% were >1.3 mm in the *BdGSc*-silenced group (Figure 3C). It appears that both *BdGSs* genes are involved in the ovarian development of *B. dorsalis* females.

**Figure 4:**
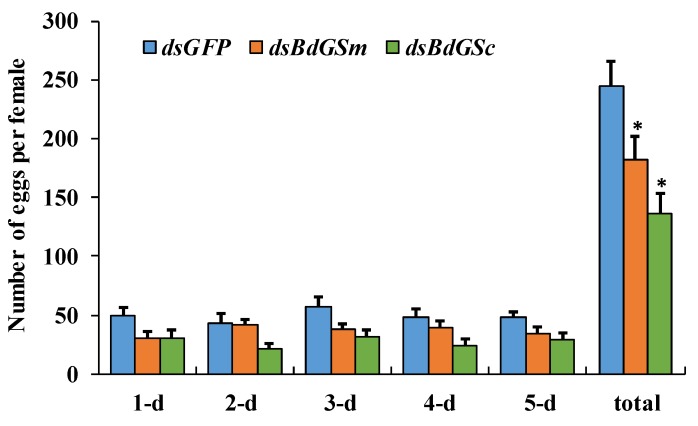
Egg-laying of females when silenced by double-stranded RNA of *BdGSs*. The bar represents the mean number of eggs (*n* = 10), and the error bar represents the positive standard error of the mean. The asterisk above the bar indicates a significant difference compared with the control as analyzed by Student’s *t*-test using SPSS 19.0 (* *P* < 0.05).

Females injected with dsRNA three times were mated with wild males and the eggs were counted for five days. There was no difference in mating success between any target gene silencing treatment and the control. The daily number of eggs in the silenced treatments was less than that in the control, but the daily counts were similar. However, the total number of the eggs over the five days of oviposition was significantly less in the silenced treatments than in the control group (Figure 4).

### 3.3. MSX Feeding

**Figure 5:**
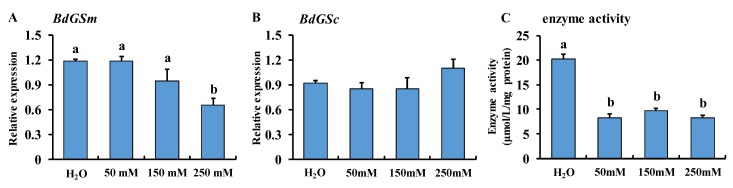
Gene expression of *BdGSm* (**A**) *BdGSc* (**B**) and enzymatic activity of glutamine synthetase (**C**) in *Bactrocera dorsalis* after feeding on the MSX inhibitor. Gene expression was calculated by qBase Plus software. The bar represents the mean gene expression and the error bar represents the positive standard error of the mean. A different letter on the bar indicates a significant difference analyzed by one-way analysis of variance with a Tukey LSD test using SPSS 19.0 (*P* < 0.05).

When females were fed a high dosage of MSX inhibitor, *BdGSm* and *BdGSc* were influenced. The expression of *BdGSm* was significantly downregulated by a high dosage of MSX, showing a dose-dependent effect (Figure 5A), while the expression of *BdGSc* was not affected by the MSX inhibitor (Figure 5B). The enzyme determination results showed that the enzyme activity was significantly inhibited at low and high dosages of the inhibitor (Figure 5C).

**Figure 6:**
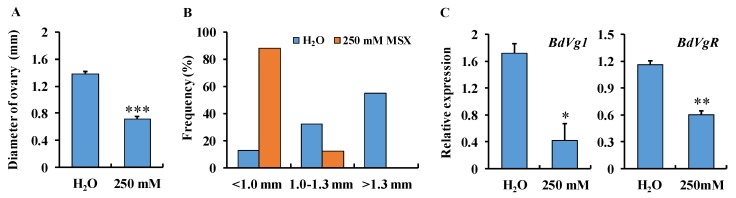
Ovarian diameter (**A**) and frequency of the ovarian size (**B**), and the expression of a *vitellogenin* and its receptor *BdVgR* (**C**) in female adults fed a high dose of 250 mM MSX. The bar represents the mean ovarian diameter (*n* = 20), frequency of each ovarian size category, and the mean gene expression, respectively. The error bar indicates the positive standard error of the mean. The expressions of two marker genes were calculated by qBase Plus software. The asterisk on the bar indicates the significant difference analyzed by a Student’s *t*-test using SPSS 19.0 (* *P* < 0.05, ** *P* < 0.01, *** *P* < 0.001).

To confirm that enzyme inhibition suppresses ovarian development, we dissected the ovaries of females fed on a high dose of MSX and measured their sizes. The ovarian diameter size of the MSX-fed females was significantly smaller than that of the control (*P* < 0.001, Figure 6A). The frequency of each subcategory differed between treatment and control group (Figure 6B). Similar to the RNAi assay, most of the ovaries in the control group were >1.0 mm, while in the MSX group, most of the ovaries were <1.0 mm. The gene expression of a *vitellogenin* (*BdVg1*) and *vitellogenin receptor* (*BdVgR*) was detected, and strong suppression of both *BdGSs* genes was observed in the high-dose MSX treatment group (Figure 6C).

## 4. Discussion

Glutamine synthetase converts glutamate and ammonia to glutamine. GS shows negligible glutamine-synthesizing activity in cells at physiological glutamine levels [3], suggesting other roles in insects. In *B. dorsalis*, GS is involved in the larval development, possibly by regulating ecdysone synthesis [15]. In the present study, we determined the expression of two *BdGSs* genes during the sexual maturation period of adults, and these genes were widely expressed at different ages of adults. In *A. aegypti*, *GS* was also constitutively expressed at all stages [8]. Different isoforms of *GSs* are expressed in a tissue- and/or development-specific manner which may be involved in distinct biological activities. In *A. aegypti*, the expression increased after a blood meal, indicating active protein synthesis in vitellogenesis [8]. It has been reported that the size of *B. dorsalis* ovary changed greatly during the development [23]. The high expression and enzyme activity of two *BdGSs* genes may also indicate high protein synthesis in female adults due to vitellogenesis, while the mitochondrial GS is mostly involved in energy metabolism. For example, in *Drosophila* embryo development, mitochondrial *GS* is highly expressed in the epidermis and in muscle [5].

Isoform differences also result in different tissue-specific expression. For instance, we found that *BdGSm* was highly expressed in the fat body, while *BdGSc* was highly expressed in the head and midgut. Tissues of fat body and midgut efficiently incorporate ammonia into amino acids using specific metabolic pathways in *A. aegypti* [24]. In the fat body, ammonia is first incorporated into the amide side chain of glutamine (Gln) and then into the amino group of glutamic acid (Glu), alanine (Ala), and proline (Pro) by a glutamine synthetase (GS) and glutamate synthase (GltS) pathway (GS/GltS pathway). By contrast, ammonia in the midgut is firstly incorporated into the amino group of Glu and Ala, and then into the amide side chain of Gln. Interestingly, the GS/GltS pathway is not functional in the midgut. GS participates in many biological processes, such as cell growth, energy metabolism, protein and nucleotide synthesis, and immune response [2]. All cells express *GS* genes but the expression varies according to developmental stage and tissues [8,25]. Similar to *BdGSs* in *B. dorsalis*, two *GSs* had different expression patterns in *D. melanogaster* tissues [5]. Surprisingly, *BdGSc* was highly expressed in the head of *B. dorsalis*. A high expression of GS has also been found in the neural tissues of *A. aegypti* [26]. This finding suggests a similar neural expression and function in *B. dorsalis* and *A. aegypti*. High expression in neural tissues and the head were also observed in *Schistocerca gregaria* and *Apis cerana* [27]. These differences between the two GS isoforms indicate their different roles in insects.

In *A. aegypti*, the GS provides the glutamine that is essential for the first step of chitin synthesis in the female *A. aegypti* peritrophic matrix [9]. Expression of *GS* in the gut can be induced by a blood meal [8]. High expression of *BdGSc* in the midgut may play a similar role in chitin synthesis. In addition to the high expression of *BdGSc* in larvae, high expression of both *BdGSs* genes was also observed in adults, especially *BdGSm* [15], indicating they have critical functions in adult stage. In the present study, we silenced target gene expression using RNAi to suppress both *BdGSs* genes. This resulted in a smaller female ovary. The significantly decrease of ovarian size indicated a developmental delay though form remained normal. Subsequent egg-laying was also significantly influenced by gene silencing, but the most likely reason was the slowed ovary development. The effect of gene suppression on egg-laying was repeatable in two independent experiments, and a stronger suppression of ovary development was found in the *BdGSc* treatment.

We inhibited enzyme activity by feeding adults the GS-specific inhibitor MSX. The enzyme activity was significantly inhibited by MSX, but the expression of *BdGSm* was only inhibited to a low transcriptional level by a high dose of MSX. The mechanism behind the decrease is unclear. MSX-induced glutamine starvation may induce amino-related energy metabolism in the fat body where *BdGSm* is highly expressed. The limited glutamine was used for basic metabolism, such as nutrition absorption and neurophysiological activity in the gut and head, where *BdGSc* is highly expressed. Hence, the expression of *BdGSc* was still at a high level comparable to the control. Similar to *BdGSc*, the *GS* expression was also not influenced by MSX injection in *N. lugens* [12]. The fat body is important for reproduction, for example, in vitellogenin synthesis [28]. When we observed the reproductive system of female flies, we found a similar delayed ovarian development. To study the reason for this reproductive inhibition, *vitellogenin* was used as an index, and it was expressed at a lower transcriptional level. The expression data showed that two *BdGSs* were differentially expressed in the tested tissues. The mechanisms behind the same downstream role and phenotype may differ in the two target gene RNAi bioassays. *BdGSm* can regulate vitellogenin synthesis in the fat body directly, while *BdGSc* can regulate ovarian development by regulating amino acid metabolism in the gut. The regulation of female fecundity by *GS* was also demonstrated in *N. lugens* [29], in which RNAi experiments changed the ovarian development of *N. lugens*. The mechanism of GS in regulating female fecundity appears to be complex. Functional analysis showed that a microRNA (miR-4868b) regulates ovarian development by targeting *GS* in *N. lugens* [11]. Gene silencing by RNAi also resulted in lower expression of the *Vg* gene in *N. lugens* [29]. A similar lower expression of *BdVg1*, as well as its receptor *BdVgR*, was also found in this study. Other research on GS in *N. lugens* showed that Gln can activate the target of rapamycin (TOR) signal pathway by promoting the serine/threonine protein kinase AKT and inhibiting 5′ AMP-activated protein kinase AMPK phosphorylation activity [12]. These findings demonstrate that GS is an important biomarker in the fecundity of female insects.

## 5. Conclusions

Previous studies indicated that glutamine synthetase (GS) plays a critical role in insects, especially in female fecundity. Two *BdGSs* are stably expressed in adult *B. dorsalis* in a tissue-specific manner. RNAi and GS-specific inhibitor bioassays delayed ovarian development and lowered egg production in adult female *B. dorsalis.* Both mitochondrial *BdGSm* and cytoplasmic *BdGSc* are involved in female fecundity. Considering their important roles in larval metamorphosis, GS genes may be targets for future insect control technologies.
